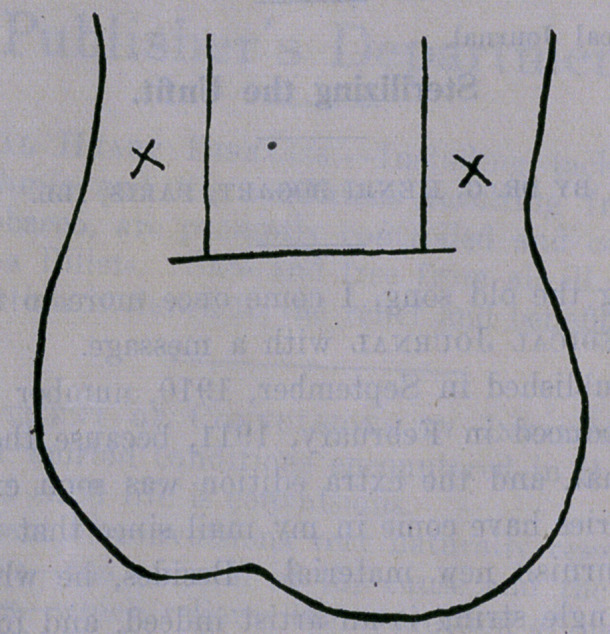# Sterilizing the Unfit

**Published:** 1912-03

**Authors:** G. Henri Bogart

**Affiliations:** Paris, Ill.


THE
### TEXAS MEDICAL JOURNAL.
Established July, 1885

F. E. DANIEL, M. D., - - - - Editor, Publisher and Proprietor

Published Monthly.—Subscription, $1.00 a Year.

VOL. XXVII. AUSTIN, MARCH, 1912. No. 9.

The publisher is not responsible for the views of the contributors.

### Original Articles.

For Texas Medical Journal.

### Sterilizing the Unfit.

BY DR. G. HENRI BOGART, PARIS, ILL.

Still singing the old song, I come once more to the readers of the TEXAS MEDICAL JOURNAL with a message.

My paper published in September, 1910, number of that JOURNAL was reproduced in February, 1911, because the demand for it was abnormal, and the extra edition was soon exhausted, and so many inquiries have come in my mail since that time that this answer will furnish new material. Besides, he who plays on a harp with a single string is an artist indeed, and to me the good that vasectomy, righteously employed, will do is one of the greatest elements of physical human uplift known to civilization.

So many have asked as to the technique of the operation, and, the text-books on surgery being silent, a plain outline of the plan is advisable.

There is a cord extending up through the anterior portion of the scrotal cavity on either side of the penis. This cord contains the *vas deferens*, the blood-vessels and the nerves of the parts.

The *vas deferens* is a tube of fibrous tissue and conveys the spermatozoa and orchitic fluid from the testicles to fertilize the semen in the seminal vesicles at the base of the bladder.

To operate, the scrotal surface is sterilized by such means as your practice has taught you to be best. The patient is best placed in an ordinary operating chair, in a semi-recumbent position. The hair should never be shaved, as the sprouting of new hair on the loose scrotal surface is exceedingly annoying. If the pubescence be excessive, it may be clipped closely. The scrotal skin at the point of operation is clasped smartly between the ball of the operator's thumb and forefinger and squeezed with a rotary motion, to produce local anesthesia. Then the spermatic cord is gently pressed into the numbed area and fixed by setting a common curved bullet forceps behind the cord.

An incision, usually three-eighths to half an inch long, parallel with the axis of the penis is made, and the *vas* is shown white and hard. Unless there have been adhesions the *vas* is free and is lifted on a curved director, and ligated to prevent a possible subsequent gonorrhea from following down the *vas* and setting up an epididymitis or orchitis. The *vas* is then cut squarely off on the testicular side of the ligature and allowed to drop back into the scrotal cavity.

Under no circumstances must the testicular end be tied, as much of the benefit results from this section of the *vas* remaining patulous and pouring the orchitic fluid, the Brown-Sequard "elixir of life," into the scrotum, to be reabsorbed by the lymphatics.

It will be well to remember that this operation is coming to be largely used as a remedial measure with violent masturbators and those suffering seminal losses and from excessive sexual perversions, to stop the drain of the vital fluid.

While only the microscope or close chemical analysis will show the change in the semen subsequently ejected, the vital fluid is retained by the patient, and a wonderful change ensues. In no case so far has there been found a closure of this open end of the *vas,* nor has there been any reported case of even temporary congestion or engorgement of any of the parts following the operation.

After the severed *vas* is dropped back into the scrotum, the wound is closed with a few drops of collodion. The operation in the hands of a deft operator usually requires about three minutes after the patient is prepared for the operation, and the patient walks from the chair without pain or inconvenience.

Of records of more than twelve hundred cases reported to me, there has not been a single untoward complication; not one has lost either desire or ability for the sexual act, and the general health and virility have been improved.

Dr. Carrington of the Virginia penitentiary, though he has no law to use it as a regulative measure, has made large use of vasectomy with that class of ignorant, vicious negroes who become morbid and malevolent through excessive masturbation, following solitary confinement, and with almost unbelievably good results.

It is well to remember that this operation is not punitive but preventive; it is not the individual subject of the operation who is the object in view, but the procreation of future descendants burdened with a heredity of crime and degeneracy and insanity to burden themselves and the community. It is well to remember that insanity has increased 183 per cent, as compared with the population's 10.0 per cent in the past two decades.

The proprietor of a private syphilitic hospital wrote me for the technique of the operation, that he might operate by consent on his cured patients, that they might not transmit the hidden taint to progeny, and this is surely for the common weal, when we remember that recent investigations show that 93 per cent of those admitted to insane asylums gave positive reactions to the Wassermann tests, though in many of these cases the virus was most certainly of hereditary taint.

The last State to join in the enforcement of this great boon to humanity—the sterilization of the unfit—is Iowa, whose law went into effect in July, 1911. This law is by long odds the best yet placed on the statute books.

Next month the readers of the Texas Medical Journal will be given an analysis of this law, together with the legal opinions of legislation thereon, as given by the Attorney General of California to the Supreme Court of that State in supporting their law, which, by the way, is the most ineffective yet placed upon the statute books.

---

For Texas Medical Journal.

## Figures and Tables

**Figure f1:**